# A new mechanism of strain transfer in polycrystals

**DOI:** 10.1038/s41598-020-66569-7

**Published:** 2020-06-22

**Authors:** F. Di Gioacchino, T. E. J. Edwards, G. N. Wells, W. J. Clegg

**Affiliations:** 1 Gordon Laboratory, Department of Materials Science and Metallurgy, The University of Cambridge, 27 Charles Babbage Rd, Cambridge, CB3 0FS UK; 2 Advanced Steel Processing and Products Research Center (ASPPRC), Department of Metallurgical and Materials Engineering, Colorado School of Mines, 1500 Illinois St, Golden, CO USA; 3 Laboratory for Mechanics of Materials and Nanostructures, Swiss Federal Laboratories for Materials Science and Technology (EMPA), Feuerwerkerstrasse 39, 3602 Thun, Switzerland; 4 Department of Engineering, The University of Cambridge, Trumpington St, Cambridge, CB2 1PZ UK

**Keywords:** Metals and alloys, Characterization and analytical techniques, Coarse-grained models

## Abstract

At the grain boundaries of plastically deforming polycrystals, strain transfer mechanisms can accommodate the shear strain carried by slip bands and mechanical twins to prevent stress build-ups and damage. So far, only the accommodation obtained through slip (and twinning) alone has been considered in the mechanism known as slip (and twin) transfer. Here, a strain transfer mechanism that also requires the rotation of the crystal lattice is demonstrated. A region of accumulated slip develops perpendicular to the active slip plane in the impinged grain. The slip gradients enable a localized lattice rotation that accommodates the shear strain in the incoming band, preventing the build-up of interfacial stresses. The mechanism operates preferentially at the boundaries between highly misoriented grains. Facilitating strain transfer at these interfaces opens up new possibilities to improve the mechanical properties of polycrystals, as discussed.

## Introduction

**Figure 1:**
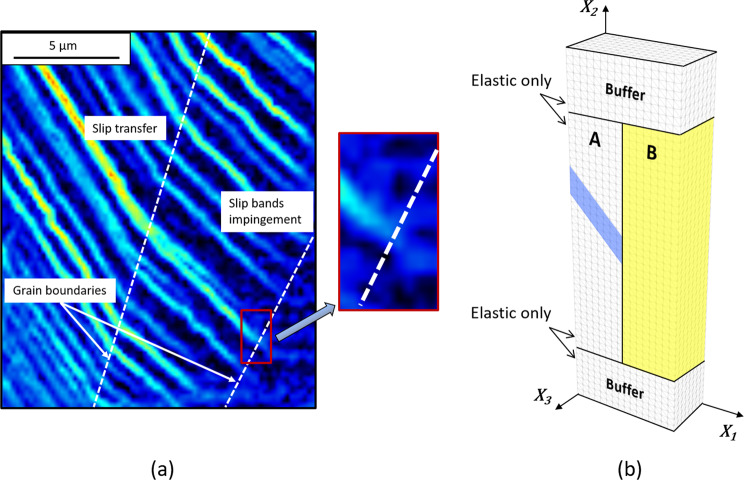
(**a**) Sub-micron resolution strain map showing instances of slip transfer and slip bands fading out at a Σ-boundary and a HAB in austenitic stainless steel, respectively. (**b**) FE model. Plastic deformation is restricted to the coloured cells.

Crystals can undergo *plastic* strain by the glide of dislocations and the cooperative, lateral displacement of rows of atoms on specific lattice planes and directions, as in slip and mechanical twinning, and by stress-induced phase transformation^1^. At the microscale, plastic strain accumulates in lamellar regions that are manifested at the surface of the deformed crystal as regularly spaced bands^2^. In a polycrystalline material, piling up of dislocations at microtructural interfaces may prevent these bands from reaching the grain boundaries, as shown in the shear strain map of a deformed austenitic stainless steel in Fig. 1a. However, the bands often appear to cross into the neighbouring grains at low angle boundaries (LABs) and Σ-boundaries^3–12^, as shown in the same figure. This mechanism of plastic strain transfer known as slip transfer (or transmission) prevents the build-up of interfacial stresses^13–16^, contributing to the higher resistance of LABs and Σ-boundaries to fatigue cracking^17–19^ and stress-corrosion cracking^20–22^ than the otherwise non-special boundaries between highly misoriented grains (HABs). The fraction of LABs and Σ-boundaries can be increased using thermo-mechanical processing methods collectively known as grain boundary engineering (GBE)^23,24^. However, only a partial improvement of damage resistance would be achieved if strain transfer is impeded at the remaining HABs^13^. The aim of the present study is then to investigate how plastic strain may transfer where slip bands (or mechanical twins) intersect grain boundaries in conditions that inhibit slip transfer.

We first consider the geometric factor −1 ≤ *N*_*LC*_ ≤ 1 introduced by Livingston and Chalmers^25^, and defined as:

1 $${N}_{LC}=({{\boldsymbol{m}}}_{A}\cdot {{\boldsymbol{m}}}_{B})({{\boldsymbol{s}}}_{A}\cdot {{\boldsymbol{s}}}_{B})+({{\boldsymbol{m}}}_{A}\cdot {{\boldsymbol{s}}}_{B})({{\boldsymbol{m}}}_{B}\cdot {{\boldsymbol{s}}}_{A})$$

where the unit vectors ***m***_*A*_ and ***m***_*B*_ are the normals to the slip planes, and ***s***_*A*_ and ***s***_*B*_ are the slip directions in the neighbouring volumes A and B. If multiplied by a pure shear stress on the slip system (***m***_*A*_, ***s***_*A*_), *N*_*LC*_ gives the shear stress resolved onto the slip system (***m***_*B*_, ***s***_*B*_). According to the authors of the same study, this factor expresses the likelihood that the activation of (***m***_*A*_, ***s***_*A*_) would contribute to (or counteract) the activation of (***m***_*B*_, ***s***_*B*_).

To illustrate how *N*_*LC*_ varies, (***m***_*A*_, ***s***_*A*_) can be kept fixed with respect to the boundary plane and the factor calculated for different orientations of (***m***_*B*_, ***s***_*B*_). For reasons thatwill be apparent in the numerical simulations presented below, we take:

2 $${{\boldsymbol{m}}}_{A}=\frac{1}{\sqrt{2}}{{\boldsymbol{e}}}_{1}+\frac{1}{\sqrt{2}}{{\boldsymbol{e}}}_{2}$$

3 $${{\boldsymbol{s}}}_{A}=-\frac{1}{\sqrt{2}}{{\boldsymbol{e}}}_{1}+\frac{1}{\sqrt{2}}{{\boldsymbol{e}}}_{2}$$

where (***e***_1_, ***e***_2_, ***e***_3_) is an orthonormal basis. Note that both ***m***_*A*_ and ***s***_*A*_ are at 45° with ***e***_2_, and have zero ***e***_3_ component. The orientation of slip plane normal ***m***_*B*_ and direction ***s***_*B*_ is instead varied and given by the values of the angles *α* and *β* as:

4 $${{\boldsymbol{m}}}_{B}=sin(\alpha )cos(\beta ){{\boldsymbol{e}}}_{1}+cos(\alpha ){{\boldsymbol{e}}}_{2}+sin(\alpha )sin(\beta ){{\boldsymbol{e}}}_{3}$$

5 $${{\boldsymbol{s}}}_{B}=-cos(\alpha )cos(\beta ){{\boldsymbol{e}}}_{1}+sin(\alpha ){{\boldsymbol{e}}}_{2}-cos(\alpha )sin(\beta ){{\boldsymbol{e}}}_{3}$$

The expression for ***m***_*B*_ (and ***s***_*B*_) can be obtained by rotating (0, 1, 0) (and (−1, 0, 0)) by *α* about −***e***_3_ and then by *β* about −***e***_2_, following the right hand convention.

**Figure 2:**
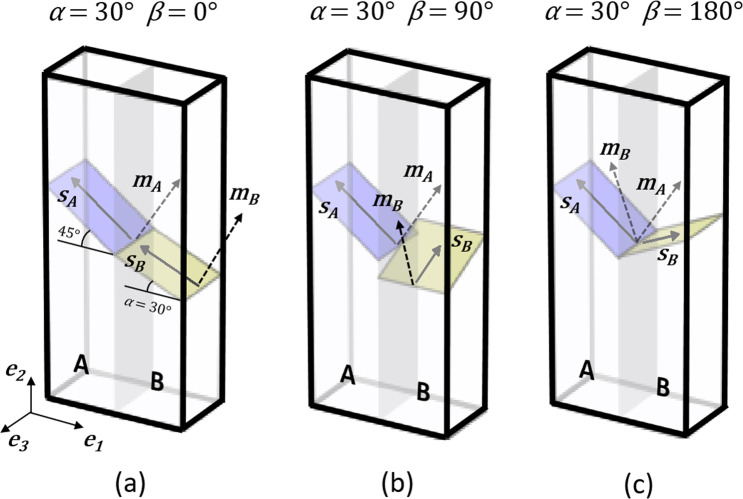
Orientations of slip planes and directions of the slip systems in neighbouring volumes A and B for which strain transfer is investigated. Whereas (***m***_*A*_, ***s***_*A*_) is fixed, the orientation of (***m***_*B*_, ***s***_*B*_) is determined by the values of the angles *α* and *β*. (**a**–**c**) Configurations for *α* = 30° with *β* = 0°, *β* = 90° and *β* = 180°, respectively.

**Figure 3:**
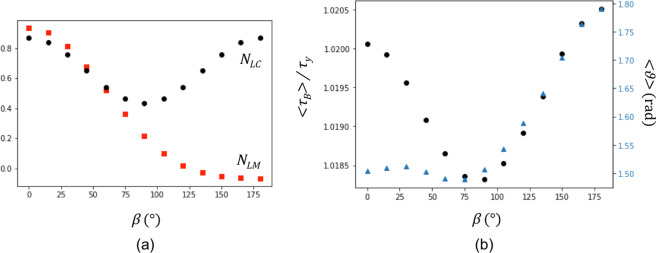
(**a**) Variation of the stress transfer factor *N*_*LC*_ and the slip transfer factor *N*_*LM*_ as a function of the angle *β*. (**b**) Black dots: variation of <*τ*_*B*_>/*τ*_*y*_ calculated at 0.01 applied strain (final step). Blue triangle markers: angle of lattice rotation averaged over the volume B also calculated at 0.01 applied strain.

The cases for *α* = 30° and *β* = 0°, *β* = 90° and *β* = 180° are illustrated as an example in Fig. 2a–c, respectively. The variation of *N*_*LC*_ with *β* for *α* = 30° is instead shown in the plot of Fig. 3a. The values range between 0.43 at *β* = 90° and 0.86 at *β* = 0° and *β* = 180°. This indicates that the activation of (***m***_*A*_, ***s***_*A*_) would contribute to the activation of (***m***_*B*_, ***s***_*B*_) for all *β*, and that such contribution would be maximum for *β* = 0° and *β* = 180°.

Although *N*_*LC*_ could be referred as a (shear) stress transfer factor, this has been used as a criterion for slip transfer in more recent studies. Poor agreement between predictions and experimental evidence has then led researchers to test alternative criteria for slip transfer, as reviewed in Bayerschen *et al*.^10^. For the purpose of comparison, the variation of the slip transfer factor ${N}_{LM}=({{\boldsymbol{m}}}_{A}\cdot {{\boldsymbol{m}}}_{B})({{\boldsymbol{s}}}_{A}\cdot {{\boldsymbol{s}}}_{B})$ by Luster and Morris^26^ with *β* (keeping *α* = 30°) is added to the plot in Fig. 3a. At *β* = 0°, the value of *N*_*LM*_ is positive and a maximum, i.e. this particular orientation of (***m***_*B*_, ***s***_*B*_) would be the most favourable one for slip transfer. As *β* increases, *N*_*LM*_ decreases, turning negative between 120° <*β* <135°, i.e. slip transfer would be progressively inhibited and eventually prevented. These predictions better agrees with the aforementioned evidence of slip bands crossing mostly LABs, as the closer the orientations of the two neighbouring grains are, the greater the chance is that the active slip systems are closely oriented to give *N*_*LM*_ ≈ 1. Similarly, the more “distant” the two orientations are, as at HABs, the greater the chance is that the active slip systems are oriented to give small values of *N*_*LM*_ and, in particular, *N*_*LM*_ <0.

**Figure 4:**
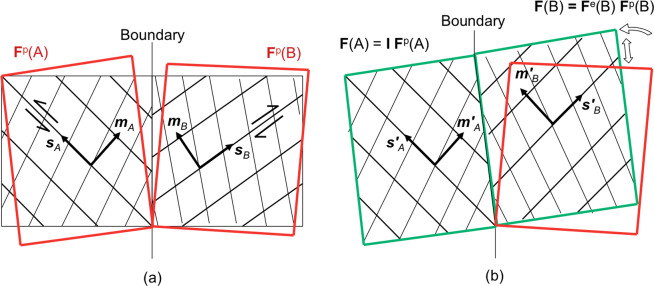
Deformation can be decomposed into a plastic and an elastic part, which includes lattice rotations, such that the deformation gradient tensor **F** is expressed as **F** = **F**^**e**^**F**^**p**^. The plastic part is given by slip only, and leads to an intermediate (imaginary) configuration in which neighbouring material elements are allowed to interpenetrate or detach. (**a**) The intermediate configuration generally presents such deformation incompatibility between neighbouring elements. Neglecting the relatively small contribution that could come from elastic strains, lattice rotation becomes necessary to maintain a compatible deformation, as in the scenario depicted in (**b**).

Here, we argue that strain transfer would also occur for 90° <*β* ≤ 180° but not as slip transfer. This is because, whenever the shear strain on the two slip systems causes interpenetration or detachment of volume elements at the boundary, in what is known as the intermediate configuration^27^, the crystal lattice can rotate locally as geometrically required^28^ to restore a compatible deformation by strain transfer, see Fig. 4. To explore how this localized lattice rotation, i.e. lattice curvature, is accomplished in what would be regarded as a distinct mechanism of strain transfer, we model the impingement of a slip band on (***m***_*A*_, ***s***_*A*_) in volume A into volume B for 0° ≤ *α* ≤ 60° and 0°≤ *β* ≤ 180°. We focus, in particular, on the mechanics of strain transfer in the conditions of deformation incompatibility described above, i.e. for 90° <*β* ≤ 180°. Experimental evidence of the foreseen mechanism is then provided by analyzing 2D nanoscale maps of plastic strain and lattice rotation in two structural materials: lamellar γ-TiAl, which has promising high-temperature lightweight applications^29^, and austenitic stainless steel. We also re-examine experimental observations found in previous studies to provide clues of the mechanism operating in other polycrystalline materials of interest, and indicate an existing geometrical factor as a basis for a potential, unifying criterion of strain transfer.

## Numerical simulations

The large strains, crystal plasticity finite element (FE) calculations in Miehe^30^ were implemented using the open-source FEniCS computing platform^31^ and employed to simulate the response of the bicrystal volume in Fig. 2 to a tensile strain of 0.01 applied along ***e***_2_ in 50 displacement increments. Details of the calculations and materials parameters are provided in the Methods section. The FE model, which contains Lagrange P_2_, tetrahedral elements in a structured mesh is instead illustrated in Fig. 1b with outlines of volumes A and B. To ensure volumes A and B be subjected to a uniform stress state during the elastic loading, elastic buffer regions were included.

As shown in Fig. 1, the FE model represents the microscopic volume of material where a slip band approaches the neighbouring grain. Although there have been reports of dislocations being reflected back on secondary slip planes at an even smaller length scale^10^, the absence of localised (secondary) slip bands in the high-resolution strain maps indicates that laminar flow is predominant in such volume. The rate-independent formulation, which models quasi-static mechanical response with the tradeoff of restricting plastic deformation to single or double slip^30^, thus appears suitable to study the mechanics of strain transfer. Furthermore, no specialised elements or rules that modulate the ease of strain transfer were adopted for the facets of the FE elements within the boundary, which can therefore be regarded as fully penetrable^32–37^. This allows for introducing a crystal plasticity formulation with a minimum amount of material parameters, and enables a qualitative study of strain transfer that is independent on the specifics of a given microstructure.

Taking ***e***_2_ as the loading direction makes the Schmid factor calculated for (***m***_*A*_, ***s***_*A*_) reach its maximum value of 0.5, causing slip to start in volume A, as intended. To produce a slip band in the latter, slip was restricted to a lamellar region, as described in the Methods section and considered elsewhere to model twin transmission in a tricrystal volume^11^. Taking ***e***_2_ as the loading direction also has the advantage of making the Schmid factor calculated for (***m***_*B*_, ***s***_*B*_) independent of *β*. This facilitates the interpretation of the results as the mechanical response can be solely attributed to differences in strain transfer for varying *β*.

Output files of displacements (from which strain components can be derived), accumulated slip, γ, angle of lattice rotation, θ, and stress components have been uploaded to an Open Science Framework (OSF) repository^38^. Here, figures only include predictions obtained at the final applied strain. To visualize the progression of each quantity of interest in the 50 time steps, the files can be downloaded and opened individually using dedicated open-source software, such as Paraview^39^.

**Figure 5:**
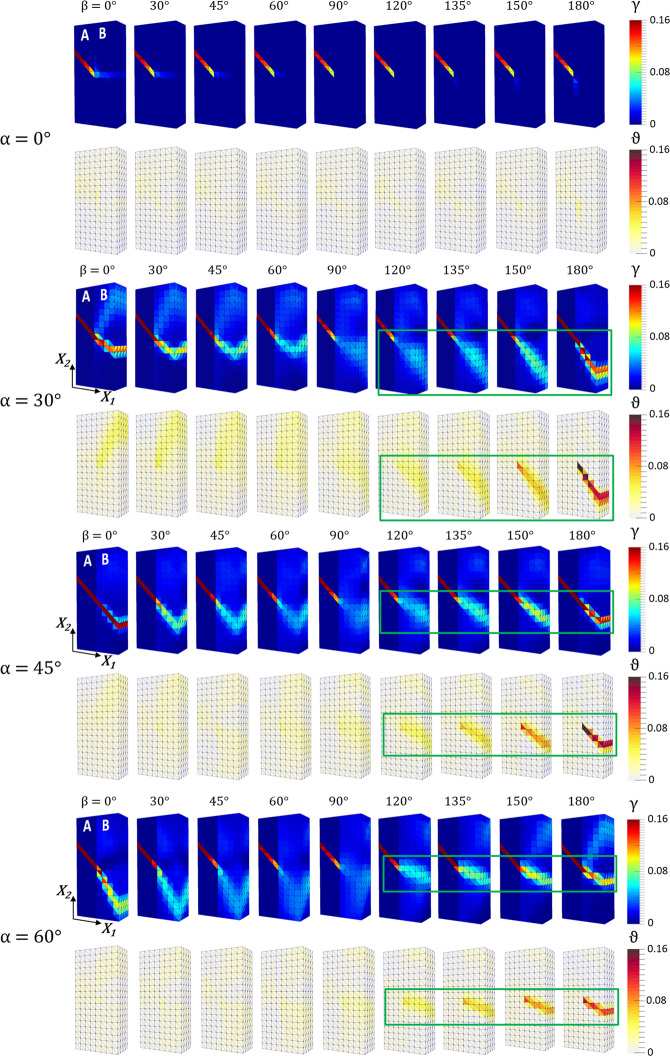
Accumulated slip γ and angle of lattice rotation θ at 0.01 tensile strain (final step) for combinations of *α* = (0°, 30°, 45°, 60°) with *β* = (0°, 30°, 45°, 60°, 90°, 120°, 135°, 150°, 180°) using a relatively coarse FE mesh. The rectangles highlight the deformation feature of accumulated slip and lattice curvature that propagates perpendicular to the slip plane in volume B from the impinging slip band in volume A.

First, a set of 36 *initial* orientations of (***m***_*B*_, ***s***_*B*_) given by combinations of *α* = (0°, 30°, 45°, 60°) with *β* = (0°, 30°, 45°, 60°, 90°, 120°, 135°, 150°, 180) were investigated using a relatively coarse mesh for computational efficiency. Predictions of the accumulated slip and the (angle of) lattice rotation at 0.01 (final step) applied strains are shown in the undeformed (material) configuration in Fig. 5.

As anticipated, *α* determines the Schmid factor in volume B. This angle was thus found to affect the ease of plastic deformation of volume B under the applied strain, Fig. 5, but not to influence the mechanics of strain transfer. The case for *α* = 30° used as example above was then chosen for in-depth analysis. Four additional orientations *β* = (15°, 75°, 115°, 165°) were considered to give intervals of 15° between 0°≤ *β* ≤180°.

**Figure 6:**
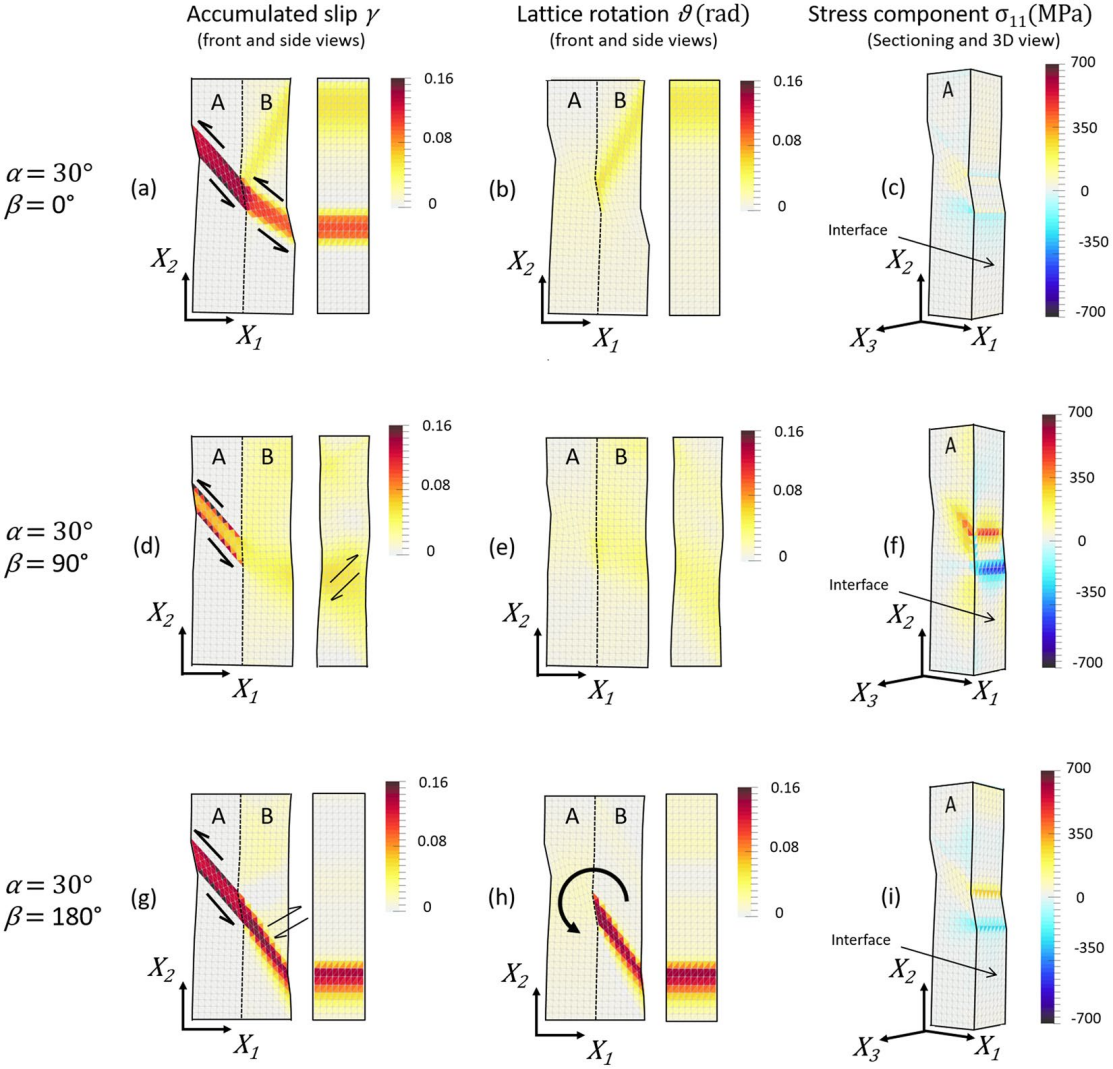
Predictions at 0.01 tensile strain (final step) for the three representative cases depicted in Fig. 2 (i.e. α = 30° and *β* = 0°, *β* = 90° and *β* = 180°, respectively). A factor of five in the displacement field is used to represent the deformed mesh. First column (a, d, and g): accumulated slip, γ. Second column (b, e, and h): angle of lattice rotation, θ. Third column (c, f, and i): stress component *σ*_11_ at the interface.

Predictions for the three representative cases in Fig. 2a–c are shown in the deformed (spatial) configuration in Fig. 6. In the first and second columns are the accumulated slip and angle of lattice rotation (front and side views), respectively. In the third column is the stress component *σ*_11_ in volume A, which is displayed on a 3D view to expose the boundary plane.

The slip band in volume A starts developing at 0.0022 applied strain. The amount of slip decreases towards the interface because the material in volume B is still deforming elastically and therefore can accommodate only a small amount of strain at the expense of the rise of a localised back stress. At larger applied strains, this stress contribution eventually causes the onset of slip in the material of volume B adjacent to the incoming slip band.

For *β* = 0°, slip in volume B takes place in a lamellar region that propagates from the area where the slip band in volume A impinges volume B. This lamellar region is parallel to the slip plane of normal ***m***_*B*_ and therefore is manifested as a slip band on the free surface of volume B, Fig. 6a. In this region, the shear strain on the slip plane reaches ~0.16 while the lattice rotation, which manly accumulates at the top half of volume B, is less than 0.015 radians (~1°), Fig. 6b. Such combination of slip band continuity across the interface and negligible lattice curvature is what characterizes slip transfer^3,40^. Predictions of a uniform *σ*_11_ field at the interface in Fig. 6c confirm that the transfer of plastic strain prevents the build-up of interfacial stresses.

For 0°<*β* ≤ 90°, strain transfer by slip transfer is still evident, although the transmitted slip band is less intense and delineated, Fig. 5. For *β* = 90°, both slip and lattice rotation in volume B are minimal, as also shown in Fig. 6d and Fig. 6e, respectively. The lack of strain transfer causes the accumulation of *σ*_11_ at the interface, Fig. 6f. Consistent with the direction of shear strain in the incoming slip band, values are positive above such band and negative below it.

For *β* > 90°, i.e. in the range of interest, a distinct deformation feature highlighted using green rectangles in Fig. 5 appears in volume B, which becomes more pronounced as *β* approaches 180°. As shown for *β* = 180° in Fig. 6g, this feature does not develop along the trace of the slip plane (of normal ***m***_*B*_) but transverse to such trace. With strain, this band thickens giving rise to a plume feature. The strong gradients of shear strain along the slip direction ***s***_*B*_ accompany a significant localized rotation of the lattice about the axis normal to both ***m***_*B*_ and ***s***_*B*_ (as geometrically required^41^ in volumes deforming by single slip under uniaxial load, and as observed experimentally in early testing of single crystals^42^). For *β* = 180°, the rotation of the lattice in the region reaches 0.16, Fig. 6h, about ***e***_3_. This rotation accommodates the shear strain in incoming slip band, as both spans an equivalent angle about the same axis, enabling the strain transfer. The visualization of the *σ*_11_ field in Fig. 6i confirms that the transfer limits the accumulation of interfacial stresses, which, although present, are much reduced in comparison with those predicted for *β* = 90°.

**Figure 7:**
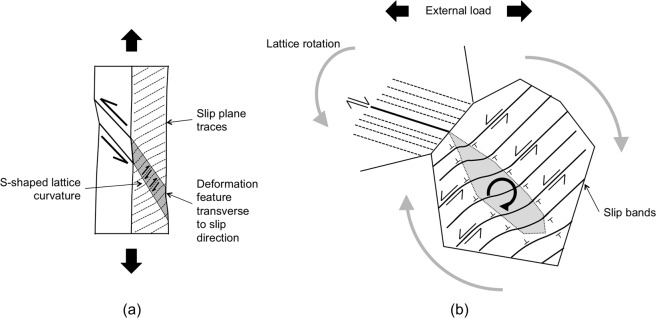
(**a**) Schematic illustrating the mechanics of plastic strain transfer as predicted in the bicrystal deformation for α = 30° and *β* = 180° in Fig. 6. A deformation feature develops perpendicular to the slip direction. In this region, accumulated slip allows for a localized lattice rotation that accommodates the shear strain in the incoming slip bands, enabling strain transfer. (**b**) Strain transfer by the same mechanism as observed in the experimental deformation mappings. The GNDs, which are indicated using the T symbol, accompany the S-shaped lattice curvature.

The kinematic of strain transfer for *β* = 180° is illustrated in Fig. 7a. Due to the general inability of crystal plasticity to capture slip banding^43^, slip in the deformation feature of localized lattice rotation will in reality distribute discretely among parallel slip bands, as illustrated in Fig. 7b. Along these bands, so-called geometrically necessary dislocations (GNDs) would accompany the (elastoplastic) lattice curvature^41,44,45^, as depicted in the same figure.

**Figure 8:**
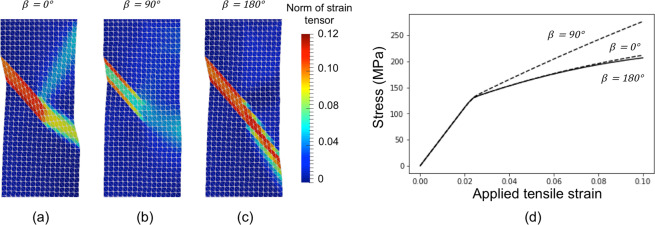
(**a**–**c**) Strain magnitude at 0.01 tensile strain for the three illustrative cases depicted in Fig. 2 (α = 30° and *β* = 0°, *β* = 90° and *β* = 180°), respectively. A factor of five in the displacement field is used to represent the deformed mesh. (**d**) Stress-strain curves for the three cases.

The norm of the Green-Lagrangian strain tensor in Fig. 8a–c helps visualize the strain transfer in a single scalar field. The corresponding stress-strain curves are plotted in the graph of Fig. 8d. As expected, strain transfer reduces the work done by the external load. In particular, the flow stress for *β* = 0° and *β* = 180° is about 20% lower than that obtained for *β* = 90°. Furthermore, the flow stress is slightly lower for *β* = 180° than *β* = 0°, as the crystal lattice at the plume rotates towards orientations that give higher values of Schmid factor.

## Experimental evidence

Recent experimental studies investigated the polycrystalline plasticity of austenitic stainless steel^40^ and lamellar γ-TiAl^46^ down to the length scale of individual slip bands and mechanical twins using the novel high-resolution digital image correlation (DIC) technique described in Methods, and electron backscatter diffraction (EBSD) to characterize the microstructure. The images used for DIC and the EBSD data can be found in the respective OSF repositories^47,48^.

These studies characterised quantitatively well-established mechanisms apt to maintain a compatible deformation between grains, such as the activation of secondary slip systems near the grain boundaries, and yet the nature of certain deformation features remained not fully understood. Here, these features are investigated in light of the mechanics of strain transfer described above. Hereafter, we refer to the in-plane components of shear strains and lattice rotations, see Methods for details.

**Figure 9:**
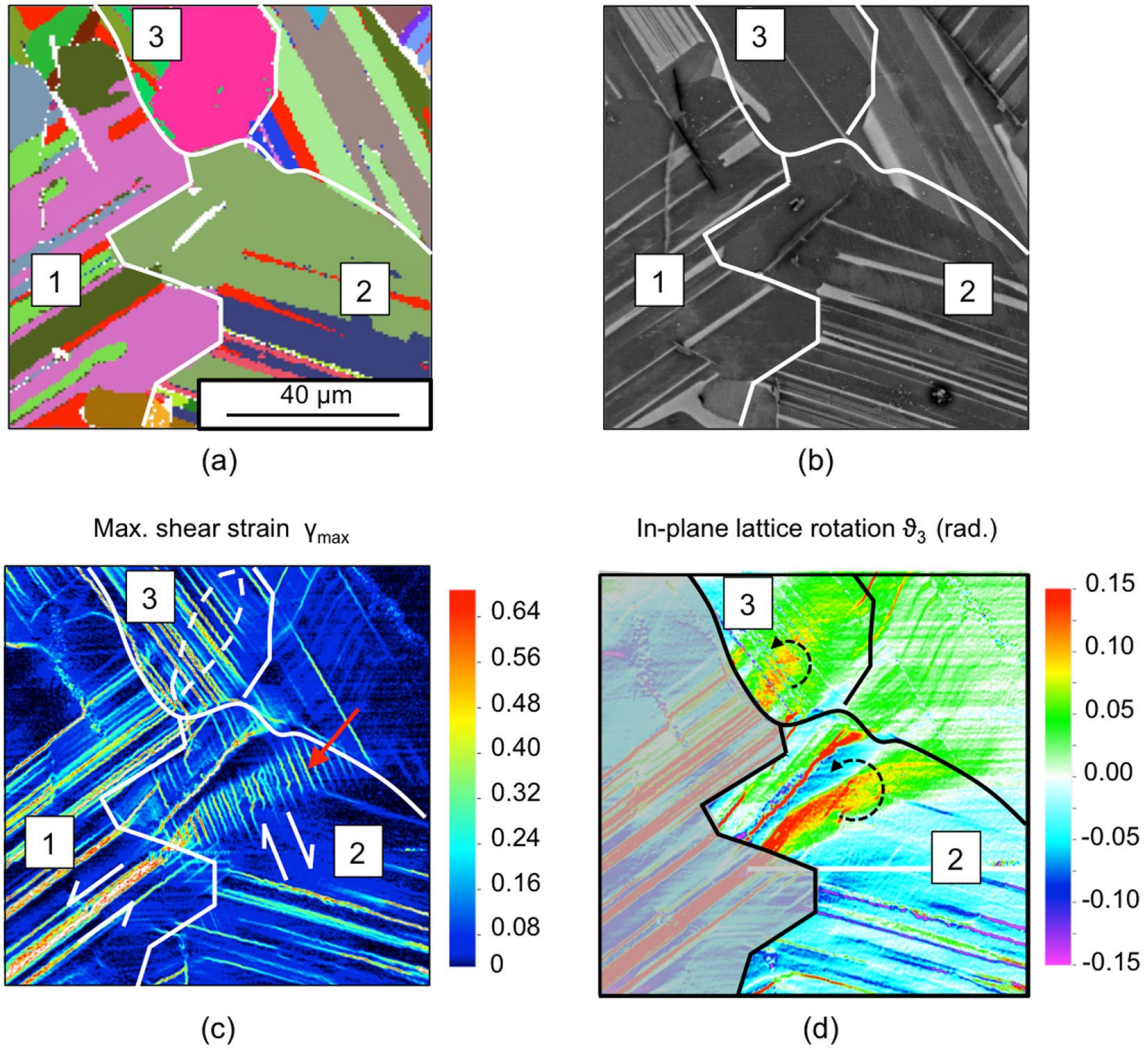
Lamellar γ-TiAl compressed at 700 °C along the vertical direction, at 3% strain. (**a**) Backscatter electron diffraction map. Red identifies *α*_2_ lamellae, other colors identify γ lamellae. (**b**) Backscatter electron imaging. Bright regions correspond to *α*_2_ lamellae. (**c**) Maximum shear strain and (**d**) in-plane lattice rotation from DIC measurements.

An instance of such deformation features is shown in Fig. 9 for the lamellar γ-TiAl. The stacks (or colonies) of soft γ and hard *α*_2_ lamellae are visible in the EBSD map in Fig. 9a and in the SE image in Fig. 9b. As shown in the maximum shear strain map in Fig. 9c, the lamellar structure tends to inhibit transverse deformation modes and hence confine slip and twinning to the lamellar planes of the γ lamellae (longitudinal mode)^49,50^.

In Stack-1, an anticlockwise (in-plane) shear strain of ~0.2 is measured in correspondence with the longitudinal twin indicated in Fig. 9d. Ahead of such twin, in the region of Stack-2 pointed to by a red arrow, tens of ~10 μm long slip bands give rise to a deformation feature that outspreads to a ~50 μm distance. This feature of accumulated slip is transverse to the trace of the slip plane, as is the deformation feature described in the numerical simulations for 90° <*β* ≤ 180°. The resulting slip gradients produce the plume-shaped feature of localized lattice rotation highlighted in Fig. 9d. The (in-plane) rotation in the proximity of the mechanical twin is also ~0.2. Moreover, it is anticlockwise as is the direction of the shear strain in the incoming twin. As illustrated in Fig. 7, this enables the strain transfer.

In Stack-2, the accumulation of slip that accompanies the localized lattice rotation is easily distinguishable to the naked eye because of the circumscribed nature of the strain transfer event. In fact, more often, it is the mapping of the lattice rotation that provides clues of strain transfer by the mechanism described here. The lattice rotation map in Fig. 9d, for instance, reveals the presence of the characteristic plume-shaped regions of localized lattice rotation also in Stack-3. In these, as in Stack-2, the in-plane rotation is anticlockwise, as is the direction of the shear strain in the incoming slip bands. The accumulation of slip in the region can then be also attributed to the transfer of strain from incoming slip bands in Stack-1, from which the plumes originate.

**Figure 10:**
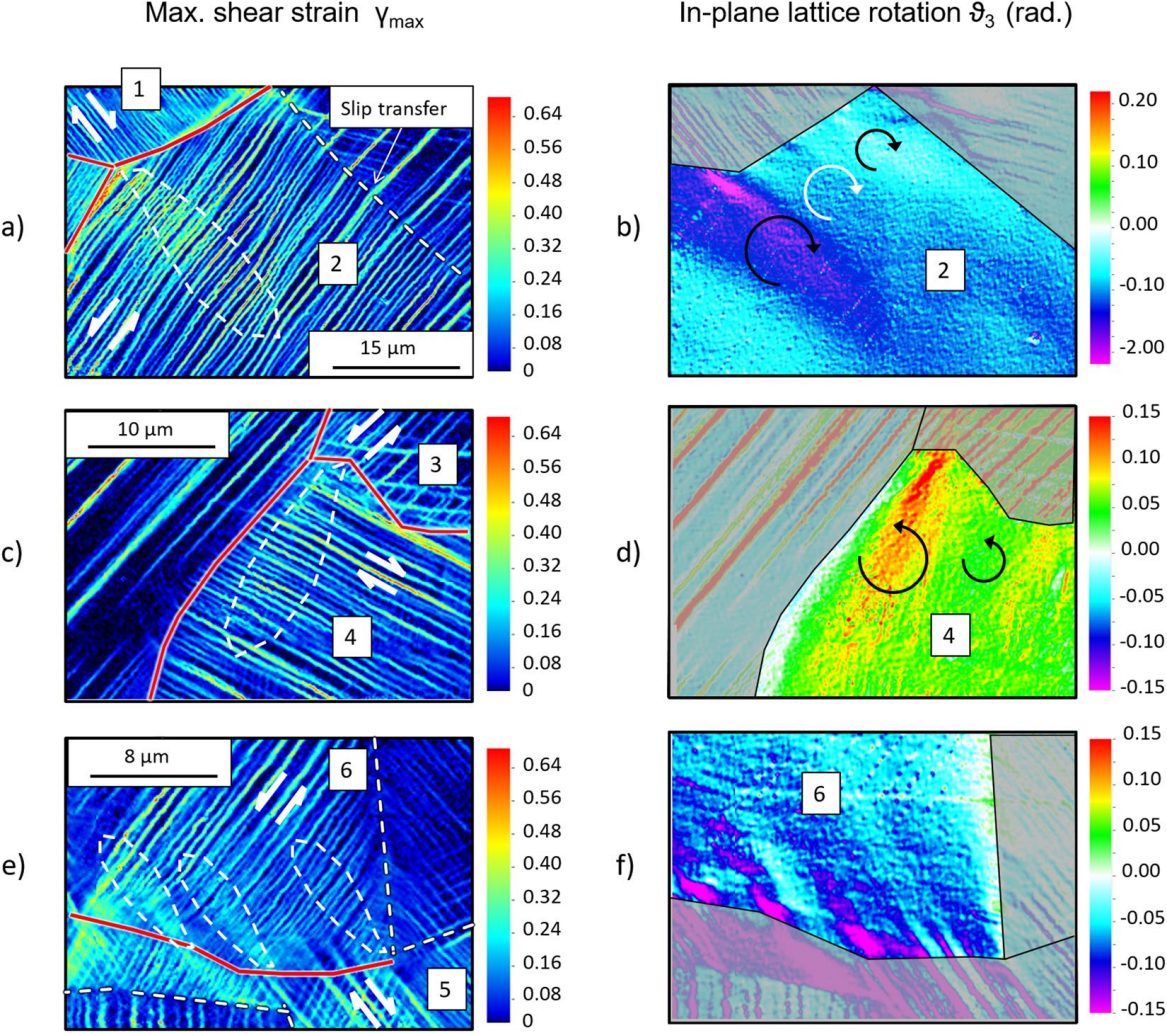
Austenitic stainless steel strained in tension at room temperature along the horizontal direction, at 6% strain. The HABs are highlighted in solid red line, LABs and Σ boundaries in dashed line. (**a**), (**c**) and (**e**) maximum shear strain. (**b**), (**d**) and (**f**) in-plane lattice rotations from DIC measurements.

Instances of similar deformation features are shown in Fig. 10 for austenitic steel. These features are found in the proximity of HABs. In Grain-1 in Fig. 10a, the shear strain along the slip bands is ~0.3 clockwise. In the neighbouring Grain-2, the direction of the shear strain is instead anticlockwise, i.e. opposite to the direction of shear in the impinging bands as obtained for 90° <*β* ≤ 180° in the numerical simulations. In Grain-2, a ~10 μm thick feature propagates transverse to the slip bands for tens of microns from the triple point where pronounced slip bands in Grain-1 meet the boundary. Although more difficult to distinguish than the one considered for the titanium aluminide, it can be seen that the number of slip bands and the strain these carry are both greater than those in the surrounding regions of Grain-2. The resultant slip gradients accompany the plume of localization lattice rotation outlined in Fig. 10b. The rotation is clockwise as is the direction of the incident slip band, once more consistent with the mechanics of strain transfer described above.

Extracting lattice rotations also reveals additional, less pronounced strain transfer events taking place along the same boundary, as highlighted in the same figure. Stress concentration at the triple point may have then favoured the accumulation of plastic strain in the incident slip bands, resulting in a more pronounced strain transfer event.

Strain transfer between Grain-3 and Grain-4 in Fig. 10c,d, and between grains Grain-5 and Grain-6 in Fig. 10e,f, is also causing the formation of similar deformation features in Grain-4 and Grain-6, respectively. In the first case, the accumulation of slip on the impinging slip band and the region of localized lattice rotation are more prominent when closer to a triple point, as observed for Grain-1 and Grain-2. By contrast, in the second case, slip accumulates on multiple and relatively wide-spaced slip bands each inducing strain transfer along the HAB.

## Clues from Previous Studies

Deformation features resembling those described here for lamellar γ-TiAl and austenitic stainless steel can be found in the optical microscopy images of a Fe-3%Si bicrystal with a symmetric HAB in Hirth^51^, in the SEM images of cyclic loaded Cu bicrystal with a HAB in Zhang and Wang^17^, and in the more recent submicron resolution digital image correlation measurements of a polycrystalline René Ni-based superalloy in Stinville *et al*.^52^ (examples of these can be found in Fig. 6, Fig. 13a and Fig. 12 in the respective studies). As observed in lamellar γ-TiAl and austenitic steel, these features propagate transverse to the slip bands in the region, and from boundaries between grains that were expected to shear in opposite directions. The plumes appear as regions of accumulated slip that, as discussed, would generate the S-shaped lattice curvature necessary to maintain a compatible deformation.

In Hirth, the formation of the plumes was mistakenly attributed to the activation of secondary slip systems, as the contribution of localized lattice rotations was not considered to illustrate how neighbouring grains can maintain a compatible deformation. In Stinville *et al*., and independently in Di Gioacchino and Fonseca^40^, the plumes were eventually revealed to be regions where slip accumulated in the primary slip system.

Localized lattice rotations ahead of impinging slip bands can been found in the EBSD measurements of deformed Ti alloys in Britton and Wilkinson^15^, Guo *et al*.^53,54^ and more recently in irradiated Zr in Thomas *et al*.^55^. In this case, the features are circumscribed to the immediate proximity of the grain boundaries and appear to have no crystallographic character (e.g. do not propagate perpendicular to the slip direction). The elastic nature of the lattice curvature has led authors to interpret these features as evidence of stress build-ups blocking slip bands at the grain boundaries. Likewise, grain-scale deformation incompatibilities could cause plume-like features of high lattice curvature (vs lattice rotation) to develop inside the grains^56^ as shown in the kernel average misorientation measurements in a CoCrFeMnNi high entropy alloy in Joo *et al*.^57^.

## Towards a Unifying Criterion of Strain Transfer

With evidence of a new mechanism of strain transfer, it would be possible to extend existing slip transfer factors to more general strain transfer factors. Towards this, it appears beneficial to relate here both predictions and experimental observations to the geometric factor *N*_*LC*_ in Eq. 1. As mentioned above, the latter can be considered a stress transfer factor, and therefore may represent a base for a unifying criterion of strain transfer.

The mean value of the accumulated slip in volume B at 0.01 applied strain was calculated and substituted in Eq. 8 to obtain an averaged measure of the shear stress component <*τ*_*B*_> acting on (***m***_*B*_, ***s***_*B*_). The variation of <*τ*_*B*_> with *β* normalized by the shear stress for yielding *τ*_*y*_ is shown in the plot in Fig. 3b. The same sinusoidal trend of *N*_*LC*_ in Fig. 3a is obtained (as expected, the localized lattice rotation towards higher values of Schmid factor causes *τ*_*B*_ to be significantly higher for *β* = 180° than *β* = 0°). This suggests that, as envisaged in Livingston and Chalmers, the impingement of slip bands (or mechanical twins) on grain boundaries can be accommodated plastically by strain transfer if the shear stress acting on the impinging band contributes to the activation of the most stressed slip system in the neighbouring grain.

The actual extent of a strain transfer episode in a polycrystal may possibly depend on factors not accounted for in *N*_*LC*_ and *N*_*LM*_, such as the orientation of the grain boundary with respect to the active slip systems. Experimental testing of bicrystal samples, in which the orientation of the boundary is known, therefore appears necessary to fully validate unifying criteria of strain transfer. Moreover, tens, if not hundreds, of cases ought to be considered for the outcome to be statistically significant^58^. Only a preliminary analysis is then proposed here by comparing the values of *N*_*LC*_ and *N*_*LM*_ calculated for the instances of strain transfer characterised above. The EBSD data were correlated with the slip traces in the nanoscale strain mappings to identify the vectors, ***m***_*A*_, ***s***_*A*_, ***m***_*B*_, ***s***_*B*_ in the neighbouring stacks and grains in Figs. 9 and 10, as described in Methods. Values of *N*_*LC*_ and *N*_*LM*_ are reported in Table 1. The values of *N*_*LM*_ are relatively small, ranging between −0.19 and 0.22, and negative in three out of five cases. On the other hand, *N*_*LC*_ is always positive, ranging between 0.37 and 0.89, consistent with the hypothesis of it being a base for a potential, unifying strain transfer factor.

**Table 1:** Values of *N*_*LC*_ and *N*_*LM*_ calculated in the investigated regions in Fig. 9 and Fig. 10 using a right-handed Cartesian coordinates (X, Y, Z) with X from left to right and Y from bottom to top.

Transfer From – To	*m*_*A*_	*s*_*A*_	*m*_*B*_	*s*_*B*_	*N*_*LC*_	*N*_*LM*_
**ST1 – ST2**	(0.42, −0.80, 0.43)	(0.61, 0.59, 0.52)	(0.90, 0.22, −0.38)	(0.18, −0.97, −0.13)	0.37	−0.02
**ST1 – ST3**	(0.42, −0.80, 0.43)	(0.61, 0.59, 0.52)	(0.80, 0.41, −0.43)	(0.19, −0.86, −0.47)	0.41	0.11
**GR1 – GR2**	(0.53, 0.67, 0.52)	(0.84, −0.51, −0.19)	(0.73, −0.45, 0.52)	(0.62, 0.10, −0.78)	0.21	0.22
**GR3 – GR4**	(−0.66, 0.63, −0.40)	(−0.70, −0.35, 0.62)	(−0.39, −0.92, 0.00)	(−0.90, 0.39, 0.15)	0.28	−0.19
**GR5 – GR6**	(−0.56, −0.70, −0.45)	(−0.80, 0.34, 0.49)	(−0.66, 0.50, 0.56)	(−0.70, −0.68, −0.22)	0.89	−0.05

## Outlook

Starting from the arguments on stress transfer by Livingston and Chalmers, the existence was postulated of a mechanism of strain transfer that requires not only slip, as in slip transfer, but also the localized rotation of the crystal lattice. Crystal plasticity finite element calculations were then carried out to predict how the resulting lattice curvature develops. Hence, experimental evidence was provided of the mechanism operating in two distinct polycrystalline materials of interest. Further clues were given of this being already encountered in earlier studies of strain transfer in other polycrystals.

Having chosen the FE domain to represent the sub-microscale volume surrounding a slip band impinging a grain boundary has justified the use of the (large strains) rate-independent crystal plasticity formulation. To extend the study to a polycrystalline microstructure, the implementation of viscoplastic crystal plasticity formulations that can account for the activation of multiple slip systems^59^ would be more appropriate. It would be no longer practical, however, to force the formation of slip bands simply by introducing elastic lamellae in the FE model. Of particular interest are then recent attempts to prompt the formation of slip bands in crystal plasticity simulations by introducing a spatial perturbation of the yield stress or the hardening parameter^43^. This approach could be extended to large polycrystalline aggregates to study the effect of strain transfer on larger-scale phenomena, such as the development of non-crystallographic deformation bands^40,60–62^. Alternatively, the use of discrete dislocation dynamic (DDD) and atomistic simulations could prove advantageous as these can inherently capture slip banding. Atomistic simulations, in particular, allow for a representation of the atomic structure of the grain boundary, which is expected to influence the strain transfer process^9,18,63,64^. However, unlike slip transfer, which can be detected at the early stage of plastic deformation as soon as dislocations piling up at the boundary appear to “transfer” past it, the mechanism of strain transfer described here would only be detectable after the density of GNDs reaches 10^13^–10^14^ m^−2^. As shown in DDD simulations of cantilever bending^44,45,65^, this occurs at relatively large imposed deformations that atomistic simulations cannot yet simulate because of prohibitive requirements of computational resources.

The complementary nature of the two mechanisms of strain transfer has been demonstrated indirectly by providing preliminary evidence of the one described here operating preferentially at HABs. For the first time, it is possible to envisage how to facilitate strain transfer at HABs. On the one hand, it may be possible to induce textures, i.e. non-random set of grain orientations in the polycrystal, that increase the probability of having HABs between grains well oriented for strain transfer. Crystal plasticity calculations of large polycrystalline aggregates may help to identify such textures and the thermo-mechanical processing methods that generate them. On the other hand, it must be possible to ease strain transfer at HABs by facilitating the accumulation of GNDs and hence the elastoplastic curvature of the crystal lattice. In materials that show a hierarchical microstructure, this might be achieved by controlling the microstructural parameters of the refined microstructure inside the grains. In the stacks of lamellar γ-TiAl, for instance, elastic bending of *α*_2_-lamellae can be expected to counteract the lattice curvature that is plastically accommodated in the γ-lamellae. This would be consistent with the increase in the number and size of strain tranfer features that has been recently observed for lamellar γ-TiAl with ultra-thin *α*_2_-lamellae^46^.

## Methods

### Numerical simulations

The finite strain rate-independent formulation by Miehe^30^ has been implemented in the solid mechanics library of the open-source FEniCS software^66^. Miehe’s study was the first to propose the use of the exponential map in the stress update algorithm, which is reported in Table 2 (elastic predictor) and Table 3 (return mapping). Note that the expression of the consistent tangent approximates the exact one, meaning that quadratic convergence rate is lost. The formulation adopts a non-linear (hyperelastic and Neo-Hookean) law to link the stress and the elastic strain of the crystal lattice. The strain energy per unit volume, *ψ*^*e*^, is then:

6 $${\psi }^{e}=\frac{1}{2}G({\rm{tr}}[{\tilde{{\bf{B}}}}^{e}]-3)+\frac{1}{2}K{(\mathrm{ln}{J}^{e})}^{2},$$

where ${\tilde{{\bf{B}}}}^{e}\equiv {\tilde{{\bf{F}}}}^{e}{({\tilde{{\bf{F}}}}^{e})}^{T}$ is the *elastic* left Cauchy–Green strain tensor, *J*^*e*^ = det(**F**^*e*^) and ${\tilde{{\bf{F}}}}^{e}$ is the isochoric contribution to the elastic part, **F**^*e*^, of the deformation gradient tensor **F**. In Eq. 6, the deviatoric and hydrostatic terms of the elastic deformation are multiplied by the shear modulus *G* and the bulk modulus *K*, respectively.

**Table 2:** Elastic predictor.

**Elastic predictor**
Given the displacement field *u*_*n*+1_ calculate **F** = **I** + ∇*u*_*n*+1_
Using ${{\bf{F}}}_{n}^{p}$ from the previous time step, get trial isochoric component of the elastic part of **F** ${\tilde{{\bf{F}}}}_{n+1}^{e\ast }={J}^{-1/3}{{\bf{F}}}_{n+1}{{\bf{F}}}_{n}^{p-1}$ with *J* = det(**F**_*n*+1_).
For the four slip systems *α* defined in the double slip model convect the reference lattice vector **M**^*α*^ and **S**^*α*^ into ${{\bf{m}}}^{\alpha \ast }={\tilde{{\bf{F}}}}^{e\ast }{{\bf{M}}}^{\alpha }$ and ${{\bf{s}}}^{\alpha \ast }={\tilde{{\bf{F}}}}^{e\ast }{{\bf{S}}}^{\alpha }$, evaluate the Schmid stress ${\tau }_{n+1}^{\alpha \ast }=G{{\bf{s}}}^{\alpha \ast }\cdot {{\bf{m}}}^{\alpha \ast }$ and, using the previous accumulated slip *A*_*n*_, calculate the yield function:
$${\phi }^{\alpha \ast }={\tau }_{n+1}^{\alpha \ast }-{\tau }_{cr}^{\alpha }({A}_{n})$$
Set tolerance ${{\epsilon }}_{tol}$ and check consistency. For all *α*,
IF $${\phi }^{\alpha \ast }\le {{\epsilon }}_{tol}$$:
Set ${\tilde{{\bf{F}}}}_{n+1}^{e}={\tilde{{\bf{F}}}}_{n+1}^{e\ast }$, ${{\bf{F}}}_{n+1}^{p}={{\bf{F}}}_{n}^{p}$, *A*_*n*+1_ = *A*_*n*_, ${\mathscr{A}}={\rm{\O }}$ and proceed to 5
ELSE:
Define group ${\mathscr{A}}$ of active system(s) *α* (and *β*) and GO to **box in** Table 3
Compute the left Cauchy (elastic) strain tensor ${\tilde{{\bf{B}}}}^{e}\equiv {\tilde{{\bf{F}}}}^{e}{({\tilde{{\bf{F}}}}^{e})}^{T}$ and the Eularian stress
$${\boldsymbol{\tau }}=G\,{\rm{dev}}[{{\bf{B}}}_{iso}^{e}]+K(ln\,{J}^{e}){\boldsymbol{I}}$$
Compute the Cauchy stress
*σ* = *Jτ* = *J*^*e*^*τ*
Compute consistent elasto-plastic tangent modulus ${{\mathbb{C}}}^{ep}={{\mathbb{C}}}_{vol}^{e}+{{\mathbb{C}}}_{iso}^{e}-{{\mathbb{C}}}_{iso}^{p}$ with
${{\mathbb{C}}}_{vol}^{{\boldsymbol{e}}}=(k+p){\bf{I}}\otimes {\bf{I}}-2p{{\mathbb{I}}}_{s}$ (${{\mathbb{I}}}_{s}$ is the fourth order spherical tensor)
$${{\mathbb{C}}}_{iso}^{e}=\frac{2}{3}Gtr({\tilde{{\bf{B}}}}^{e})\left[{{\mathbb{I}}}_{s}-\frac{1\,}{3}{\bf{I}}\otimes {\bf{I}}\right]-\frac{2\,}{3}[{\rm{dev}}[{\boldsymbol{\tau }}]\otimes {\bf{I}}+{\boldsymbol{I}}\otimes {\rm{dev}}[{\boldsymbol{\tau }}]]$$ $${{\mathbb{C}}}_{iso}^{p}=\sum _{\alpha \in A}\sum _{\beta \in A}{J}^{\alpha \beta -1}G\,{\rm{dev}}[{{\bf{s}}}^{\alpha \ast }\otimes {{\bf{m}}}^{\alpha \ast }+{{\bf{m}}}^{\alpha \ast }\otimes {{\bf{s}}}^{\alpha \ast }]\otimes G\,{\rm{dev}}[{{\bf{s}}}^{\beta \ast }\otimes {{\bf{m}}}^{\beta \ast }+{{\bf{m}}}^{\beta \ast }\otimes {{\bf{s}}}^{\beta \ast }]$$

**Table 3:** Return mapping algorithm.

**Return mapping algorithm and consistent elastoplastic moduli**
Set initial values Δ*γ*^*α*^ = 0 for all $\alpha \in {\mathscr{A}}$ compute
$${\tilde{{\bf{P}}}}^{e}=\exp [-\sum _{\alpha \beta \in {\mathscr{A}}}\,\Delta {\gamma }^{\alpha }{{\bf{S}}}^{\alpha }\otimes {{\bf{M}}}^{\alpha }]$$
$${A}_{n+1}={A}_{n}+\sum _{\alpha \beta \in {\mathscr{A}}}\Delta {\gamma }^{\alpha }$$
$${\tilde{{\bf{F}}}}_{n+1}^{e\ast }\,:={\tilde{{\bf{F}}}}_{n+1}^{e\ast }\,{\tilde{{\bf{P}}}}^{{\rm{e}}}$$
$${{\bf{m}}}^{\alpha \ast }={\tilde{{\bf{F}}}}^{e\ast }{{\bf{M}}}^{\alpha }$$ and $${{\bf{s}}}^{\alpha \ast }={\tilde{{\bf{F}}}}^{e\ast }{{\bf{S}}}^{\alpha }$$
$${J}^{\alpha \beta }=\,G\,({{\bf{s}}}^{\alpha \ast }\otimes {{\bf{M}}}^{\alpha }+{{\bf{m}}}^{\alpha \ast }\otimes {{\bf{S}}}^{\alpha }):[{\tilde{{\bf{F}}}}_{n+1}^{e\ast }\cdot {{\bf{D}}}_{e}:({{\bf{S}}}^{\alpha }\otimes {{\bf{M}}}^{\alpha })]-{\partial }_{A}{\tau }_{cr}$$
with **D**_*e*_ representing the derivative of the exponential map at $-\sum _{\alpha \beta \in {\mathscr{A}}}\,\Delta {\gamma }^{\alpha }{{\bf{S}}}^{\alpha }\otimes {{\bf{M}}}^{\alpha }$.
Update the plastic parameters
$$\,\Delta \,{\gamma }^{\alpha }\cdot \Delta \,{\gamma }^{\alpha }-\sum _{\alpha \beta \in A}{J}^{\alpha \beta -1}{\phi }^{\beta }$$
Calculate $${\phi }^{\alpha \ast }$$
IF ${\phi }^{\alpha \ast } < {{\epsilon }}_{tol}$ and $\Delta \,{\gamma }^{\alpha } > 0$ convergent solution, EXIT
ELSE: go to 1.

The lattice rotation **R**^*e*^(*ϑ*, *r*), where$\,\vartheta$ and $r$ are the angle and axis of rotation, respectively, is obtained from the polar decomposition of **F**^*e*^. The latter is calculated as **F**^*e*^ = **FF**^*p−*1^, where **F**^*p*^ is the plastic part of **F**, and which is given by a sum of shear contributions *γ*^*α*^ of the active slip systems (***m***^*α*^, ***s***^*α*^). In the present study, in which the crystals yield along a single slip plane, ***m***, and direction, ***s***, it is:

7 $${{\bf{F}}}^{p}={\bf{I}}+\gamma {\boldsymbol{s}}\otimes {\boldsymbol{m}}$$

The shear strain on such slip system, *γ*, which therefore coincide with the accumulated slip, appears in the hardening law used to update the value of the shear stress on the slip systems that is required to maintain slip, *τ*:

8 $$\tau ={\tau }_{{\rm{y}}}+{\tau }_{\infty }\left(1-\exp \left(-\frac{\gamma }{{A}_{0}}\right)\right)+h\gamma $$

where *τ*_c_ is the critical shear stress at which slip initiates, i.e., when *γ* = 0.

The values of these elastic constants and hardening parameters in Table 4 are taken from Miehe’s study.

**Table 4:** Values of elastic constants, yield stress and hardening parameters used in the crystal plasticity calculations (taken from Miehe^30^).

Bulk Modulus ***K***	49980	(MPa)
Shear Modulus ***G***	21100	(MPa)
Shear Stress for Yielding ***τ***_**y**_	60.0	(MPa)
Saturation stress ***τ***_***∞***_	48.0	(MPa)
Saturation Parameter ***A***_***0***_	0.929	—
Linear Hardening ***h***	1.0	(MPa)

The outputs of the displacement field, accumulated slip, lattice rotation, and stress components have been stored in a format that can be read in the open-source visualization tool Paraview^39^. This gives the possibility to extract the Green-Lagrangian strain tensor **E** = 0.5(**F**^T^**F** − **I**) from the displacement field and calculate its norm.

### Experimental

The TiAl alloy, Ti-45Al-2Nb-2Mn(at%)-0.8 vol% TiB_2_ (Ti4522XD), was received in a post-centrifugal casting hot isostatically pressed at 1250 °C, 170 MPa for 4 hours and then at 1010 °C, 140 MPa for 8 hours to obtain a nearly lamellar microstructure. The austenitic stainless steel, Fe-0.021C-0.34Si-1.96Mn-18.15Cr-9.17Ni-0.031P-0.027S, was received in the solution-annealed condition (1h at 1050 °C and water quenched).

The tensile testing of the austenitic steel was done on a flat dog-bone shaped specimen with a 5 mm gauge length using a Zeiss-Kammrath micro-tester at constant strain rate of 4 × 10^−3^ s^−1^. For the γ-TiAl, compression of 5 mm cuboid and tension of a flat dog-bone shaped specimen were carried out at a strain rate of 10^−3^ s^−1^ using a 25kN screw machine (Tinius Olsen, UK). The samples were heated in air by a halogen lamp hoop heater (Heraeus Noblelight GmbH, Germany) and 700 °C.

Before testing, the surface of both specimens was covered with a nano-scale speckle pattern, using the gold remodelling procedure described in previous studies^61,67^. At each strain level the test was interrupted, the specimen was unloaded and removed from the testing machine and mounted on the SEM stage for image acquisition.

The commercial software DaVis (LaVision, Germany) was used to carry out DIC analysis of the images in the deformation sequence using adaptive, FFT based cross-correlation with an interrogation window. The latter was of 8 × 8 px^2^ for austenitic steel and 16 × 16 px^2^ for TiAl, corresponding to 0.17 × 0.17 µm^2^ and 0.16 × 0.16 µm^2^, respectively.

The DIC analysis provides dense maps of in-plane displacement, *u*_*ij*_, which can be used to calculate the strain components, *ε*_*ij*_, that describe the in-plane strain. The maximum shear strain, γ_*max*_, used here to quantify the accumulated strain, is a scalar valued function of these components:

9 $${{\rm{\gamma }}}_{max}=2\,\sqrt{{\left(\frac{{\varepsilon }_{11}-{\varepsilon }_{22}}{2}\right)}^{2}+{\left(\frac{{\varepsilon }_{12}}{2}\right)}^{2}}.$$

The in-plane component of the lattice rotation in region of single slip deformation is extracted from DIC measurements using the method demonstrated in Di Gioacchino and Clegg^68^. Taking *X*_*sb*_ as the direction of the slip bands in such region and *u*_*n*_ as the component of the displacement normal to *X*_*sb*_, the lattice rotation is given by:

10 $${\vartheta }_{3}=\frac{\partial {u}_{n}}{\partial {X}_{sb}}\,$$

After testing, colloidal silica (OPS) was used to remove the speckle pattern and at the same time polish the surface of the samples for electron backscatter diffraction (EBSD). Lattice orientation maps of the same region were acquired with spatial resolution comparable to those of the DIC measurements using EBSD systems by Oxford Instruments (UK). The EBSD measurements were used to identify and characterize phases and boundaries in both materials, and also in conjunction with the strain maps to obtain the active slip systems. In particular, the (111) plane with trace on the plane of investigation that most closely aligned with the microbands in the strain maps was taken as the active plane. The slip or twinning direction in such plane was identified as the one of highest Schmid factor within the (1–10) directions in the austenitic stainless steel, and <1–10] ordinary dislocations, <−101] super-dislocations, and twinning on <11-2] directions of the γ-phase in the titanium aluminide.
